# Experiments on Animals

**Published:** 1888-12-08

**Authors:** 


### EXPERIMENTS ON ANIMALS.

Important observations have been made on certain animals at the Zoological Gardens in connexion with the subject of rickets. The young of carnivorous animals and of monkeys born at the "Zoo" have for years suffered exceedingly from this disease, and very large numbers have died. In the case of lions it has, until quite recently, been impossible to rear them there on account of their extreme liability to the disease. With the exception of a single litter suckled by the dam about ten years ago, the cubs invariably died until the year 1886, the cause of death being marked "rickets." Those in charge have naturally been most anxious to discover the whole of the circumstances which resulted in these losses and misfortunes and to bring about a more healthy condition of things. Mr. Bland Sutton, honorary pathologist to the Zoological Society, and Mr. Bartlett, the superintendent, have taken intelligent pains, and their efforts have at length been rewarded. Taking the case of monkeys first, the observers have become convinced that the young, when removed early from the mothers, and fed on vegetable diets, chiefly fruits, have developed rickets solely in consequence of their limitation in diet. Such diet is deficient in three essential elements, viz., proteids, animal fats, and earthy salts. Young lions, which of course have never been fed on vegetables—have, however, as has been stated, also shown a marked tendency to rickets when not suckled. Their food has, until recently consisted largely of old horse. But this, though plentiful in proteids, was deficient in fats; because, as everyone knows, "old horse" and "lean horse" are generally synonymous terms. Such food is also wanting in earthy salts; for, although the bones of old horses contain plenty of such salts, yet they are so hard that not even the teeth of the old lions, much less those of the young ones, are able to grind them. The most marked case of all was that of two young bears. These were fed on a mixed vegetable and animal diet, consisting largely of rice and biscuits, with a smaller proportion of lean meat. In this case, fat, which ought to have constituted a quarter of the full dietary, was almost entirely excluded. The bears died of extreme rickets long before reaching maturity. These facts are all more or less negative in character. The following is as decidedly positive: A lioness gave birth to a litter of several cubs. As is usual, she had very little milk from the first, and none at all at the end of a fortnight. The cubs were then put on horseflesh according to custom. They quickly developed rickets, also according to custom; and one of them died. Then, at Mr. Bland Sutton's suggestion, the diet was changed. The horseflesh was continued, but there were given in addition cod-liver oil and pounded bones with milk. No other change of any kind was made. The animals were kept in the same dens, with exactly the same amount of air, light, and warmth as before. In three months all signs of rickets had disappeared; and now, at the end of nearly two years, they are perfectly strong, healthy, and well developed. Such an event was previously unknown in the history of the society. The present writer could record more than one case of children in his own practice in which the results of a similar dietary have been quite as striking as these. The public should be careful how they allow themselves to be led away by dietetic fads and fallacies from those general lines of feeding which have borne the test of hundreds of years of experience, and the wisdom of which the observations of scientific experts from time to time so convincingly confirm.

